# Concentration and Community of Airborne Bacteria in Response to Cyclical Haze Events During the Fall and Midwinter in Beijing, China

**DOI:** 10.3389/fmicb.2018.01741

**Published:** 2018-07-31

**Authors:** Weilin Li, Jinshui Yang, Daizhou Zhang, Baozhen Li, Entao Wang, Hongli Yuan

**Affiliations:** ^1^State Key Laboratory of Agrobiotechnology and Key Laboratory of Soil Microbiology, Ministry of Agriculture, College of Biological Sciences, China Agricultural University, Beijing, China; ^2^Faculty of Environmental & Symbiotic Sciences, Prefectural University of Kumamoto, Kumamoto, Japan; ^3^Departamento de Microbiología, Escuela Nacional de Ciencias Biológicas, Instituto Politécnico Nacional, Mexico City, Mexico

**Keywords:** haze, bioaerosols, airborne bacteria, concentration, community composition, meteorological factors

## Abstract

Since 2013, severe haze events frequently have occurred in Beijing between October and March, which have created a significant public health threat. Although variations in the chemical composition of these haze events have been studied widely, information pertaining to airborne bacteria in such haze events remains limited. In this study, we characterized the concentration, community structure, and composition of the airborne bacteria in response to nine haze events that occurred between October 1, 2015, and January 5, 2016. We also analyzed the correlations of airborne bacteria (concentration, community structure, and composition) with pollution levels and meteorological factors. The results indicated that airborne bacterial concentration showed a positive cyclical correlation with the haze events, but the bacterial concentration plateaued at the yellow pollution level. In addition, we found particulate matter (PM_10_) and relative humidity to be key factors that significantly affected the airborne bacterial concentration and community structure. Moreover, *Halomonas* and *Shewanella* were enriched on haze days for all nine of the haze events. Finally, the correlations between haze pollution and airborne bacteria in midwinter were weaker than those in fall and early winter, indicating an obvious staged distinction among the effects of haze on airborne bacteria. Our study illuminated the dynamic variation of bioaerosols corresponding to the cyclical haze events and revealed the interactions among air pollution, climate factors (mainly relative humidity), and airborne bacteria. These results imply that different strategies should be applied to deal with the potential threat of airborne bacteria during haze events in different seasons.

## Introduction

Since January 2013, severe haze events frequently have occurred in Beijing (Ouyang, 2013), which have posed a serious threat to public health (Guan et al., 2016) and have disturbed the outdoor activities of the local people (Quan et al., 2014). These haze events either from primary source emissions (Wu et al., 2016) or from secondary formation (Huang et al., 2014; Wang et al., 2016) were characterized by a sharp rise in PM. The PM in the atmosphere is a complex mixture including abiotic and biological particles. Most recent studies about haze events focused on abiotic pollutants, including their chemical compositions, sources, evolutionary patterns, and potential threats to human health (Sun et al., 2013; Huang et al., 2014; Guan et al., 2016; Wang et al., 2016). Studies on the effects of haze events on bioaerosols, however, are limited. Bioaerosols refer to the biological particles, including bacteria, fungal segments or spores, and viruses that are released from soil or water bodies to the atmosphere (Fröhlich-Nowoisky et al., 2016). Bioaerosols are crucial components of aerosols because the microorganisms directly affect human health by disseminating allergens and pathogens (Griffin, 2007) and contribute to changes in the global climate by serving as biogenic ice nuclei (Bowers et al., 2009). Thus, studies on the relationships between bioaerosols and haze events are necessary to prevent their potential hazards.

The relationships between bioaerosols and haze events may be reflected by variations in airborne bacteria during haze events in both their concentration and community structure. In terms of airborne bacterial concentration (*C*_ab_), different trends during haze events have been reported, including an increase in and positive correlation with the main pollutants in several studies (Li et al., 2015; Dong et al., 2016; Wei et al., 2016), and a decrease in some other reports (Gao et al., 2015, 2016; Xu et al., 2017). This divergence in findings may be caused by the differences in both counting methods and sampling conditions. First, culture-dependent methods were used to enumerate *C*_ab_ in most of the studies (Alghamdi et al., 2014; Gao et al., 2015, 2016; Li et al., 2015), which might underestimate the bacterial concentration and cause a divergence in counting results because most of the bacteria in the environment is not culturable. In addition, the ability to count culturable bacteria also can be affected by culture medium types and conditions. Second, culture-independent methods, such as real-time quantitative PCR (Liu et al., 2017; Xu et al., 2017), sensor-ultraviolet aerodynamic particle spectrometer (Wei et al., 2016), and fluorescent dye techniques (Dong et al., 2016) were used in several studies to evaluate *C*_ab_ during the haze events, and the results of these studies were not comparable because the samples were collected from different regions and at different haze pollution levels. Haze events are cyclical processes with pollutant concentrations gradually increasing and decreasing. Almost all of the previous studies, however, only compared *C*_ab_ in individual samples collected from non-haze or haze days rather than continuously observing these parameters during the haze process (Li et al., 2015; Dong et al., 2016; Liu et al., 2017; Xu et al., 2017). Therefore, an understanding of the dynamic variation in airborne bacterial concentrations throughout the haze process remains limited.

Previous studies have shown that bacteria were the dominant airborne microorganisms in the PM (Cao et al., 2014; Zhai et al., 2018). Li et al. (2015) found that *Staphylococcus* and *Micrococcus* were the most abundant culturable bacteria on haze and non-haze days, respectively. Cao et al. (2014) demonstrated that airborne bacteria in the PM mainly were categorized as terrestrial microorganisms, and *Geodermatophilus obscurus* was the dominant species revealed by metagenomic methods. Wei et al. (2016) and Du et al. (2017), however, did not find significant difference in airborne bacterial composition between haze and non-haze days by analyzing the 16S rRNA gene sequences. But beyond that, the information related to airborne bacterial community structure and composition during haze events is limited. Moreover, although relative humidity, carbon monoxide, and ozone concentrations have been identified as the main factors affecting the diversity of bacteria in PM (Liu et al., 2017), few studies have examined variations in airborne bacterial community structure and composition responding to environmental factors in haze pollution. To date, key environmental factors that shape the airborne bacterial community remain unclear.

To explore the interactions among air pollution, climate factors, and airborne bacteria during different haze stages and at various pollution levels, in this study, samples were collected from nine haze events that occurred between October 1, 2015, and January 5, 2016. Changes were analyzed in the concentration and community structure of the airborne bacteria throughout the haze process. Additionally, the key environmental factors affecting the airborne bacteria during these haze events were researched.

## Materials and Methods

### Sample Collection and Pollution Episodes

Aerosol samples were collected from the roof of the Center of Life Sciences (CLS) Building (40°01′28″N, 116°16′41″E, ∼20 m above ground) and a balcony of the Horticultural Building (40°01′22″N, 116°16′32″E, ∼20 m above ground) at the China Agricultural University, Beijing, during the same haze event cycle. These buildings are located outside the fifth ring roads northwest of Beijing, a region that does not have a hospital, factory, dumpsite, or sewage treatment plant nearby. According to the cyclic haze events, we collected samples between October 1, 2015, and January 5, 2016, which covered nine haze cycles.

Samples collected from the Horticultural Building were used to determine the airborne bacterial concentration. Sterilized 0.2 μm polycarbonate filters (Model GTTP4700, Merck Millipore Ltd., Carrigtwohill, Cork, Ireland) in Filter Holders (Model XX4304700, Merck Millipore Ltd.) were used to sample with a flow rate of 16 × 10^-3^ m^3^/min for 1–2 h. A filter without a sample was used as the control. To avoid contamination, all tools used for replacement of filter membranes were wiped with 75% ethanol. After sampling, the filter was immediately transferred to 20 mL sterilized phosphate buffered saline (PBS, pH 7.4) at the bench-top, and the bacteria on the filter were transferred by using an ultrasonic cleaner (180 W, 15 min) in PBS for subsequent bacterial concentration detection.

Samples collected from the CLS Building were used to estimate the airborne bacterial composition. A high-volume air sampler (Model KB-1000, Qingdao Genstar Electronic Technology Co., Ltd., Qingdao, China) equipped with a fiberglass filter was used at the sampling site at a flow rate of 1.03 m^3^/min. We sterilized the filters wrapped in aluminum foil for 5 h at 200°C. The filter holder was sterilized with 75% ethanol before sampling. Sampling generally lasted 8 h except for two samples that lasted 7.5 and 7 h, respectively, according to the duration of the haze event. A sterilized filter inside a holder without sampling was used as the blank control. All samples were stored at -80°C for downstream analysis.

Hourly concentrations of PM (PM_2.5_ and PM_10_) and gaseous pollutants, including sulfur dioxide (SO_2_), nitrogen dioxide (NO_2_), ozone (O_3_), and carbon monoxide (CO), were recorded from the Beijing Municipal Environmental Monitoring Center^1^. Meteorological parameters, including temperature (T), relative humidity (RH), and wind speed (WS), were obtained from the China Weather Network^2^. Air pressure (AP) data were taken from the National Meteorological Center^3^. Detailed data are provided in Supplementary Figures S1, S2.

All samples were divided into five groups [clean, slightly polluted (SP), yellow, orange, and red] from non-haze to severe haze according to the research of Zheng et al. (2016), which considered the PM_2.5_ index and RH: when PM_2.5_ index was less than 35 μg/m^3^, the air quality was at a clean level; when the PM_2.5_ index was greater than 35 μg/m^3^, RH was considered in air quality assessment. When RH was greater than 80%, SP, yellow, orange, and red pollution levels corresponded to PM_2.5_ at 35–115, 115–150, 150–250, and >250 μg/m^3^, respectively. When RH was less than 80%, SP, yellow, orange, and red levels represented PM_2.5_ at 35–150, 150–250, 250–500, and >500 μg/m^3^, respectively (Zheng et al., 2016).

### Analysis of the Airborne Bacterial Concentration

The bacteria in PBS were enumerated by referring to the previously published method using the LIVE/DEAD^®^ BacLight^TM^ Bacterial Viability Kit (Hara and Zhang, 2012; Yuan et al., 2016). The 20 mL PBS containing air samples or a negative control were stained with 200 μL BacLight^TM^ and were incubated for 15 min in the dark at 4°C. Then this stained sample was divided into three subsamples. Bacteria in each subsample were transferred onto a 0.2 μm black polycarbonate membrane (25 mm diameter, ADVANTEC^®^, Toyo Roshi Kaisha, Ltd., Tokyo, Japan) by filtration. The total number of bacteria in 20 random regions (1 cm × 1 cm) in each membrane was counted using a fluorescent microscope under 479–490 nm excitation with 400× magnification. According to a previous study (Yuan et al., 2016), viable bacteria emitted green and yellow fluorescence, and non-viable bacteria emitted orange and red fluorescence. Then, the concentration of viable bacteria in the air was calculated as the following the formula of Dong et al. (2016) but with a few revisions:

(1) $$\begin{array}{c} C_{\mathrm{ab}}\;=\;\frac{Ave\times Mag^{2}\times S1\times V1}{S2\times V2\times V3}(1) \end{array}$$

where:

Ave: average number of viable cells in each count plate (cells),

S1: area of black polycarbonate membrane (cm^2^),

S2: area of counting chamber in eyepiece (cm^2^),

V1: volume of phosphate buffered saline (mL),

V2: volume of subsamples (mL),

V3: volume of collected air for sample (m^3^), and

Mag: magnification factor.

The very low amount of non-viable bacteria in the haze events was counted (Supplementary Figure S7). The causes of airborne bacterial death are complex, therefore no clarification about this aspect could be given in this study and data of non-viable bacteria were not further analyzed in the subsequent analyses.

### DNA Extraction and High-Throughput Sequencing of Bacterial 16S rRNA Genes

To obtain high-quality metagenomic DNA, the previously reported methods were used by applying a MO BIO PowerSoil^®^ (MO BIO Laboratories, Carlsbad, CA, United States) DNA isolation kit and a GenMag Spin^®^ (GenMag Biotechnology, Beijing, China) Viral DNA Kit (Jiang et al., 2015) in this analysis. Airborne samples on filter surfaces were scraped and transferred to PowerBead Tubes (provided by the MO-BIO PowerSoil DNA isolation kit). Before column purification, DNA was extracted according to the manufacturer’s instructions for the MO-BIO PowerSoil DNA isolation kit. To improve the quality of extracted metagenomic DNA, the column purification was replaced with magnetic bead purification using a GenMag Spin^®^ Viral DNA Kit. The blank control sample undergo the same procedure. Extracted metagenomic DNA was stored at -20°C for subsequent analysis.

For high-throughput sequencing, V3 and V4 regions of the 16S rRNA genes were amplified from the obtained metagenomic DNA with specific primers 341F (5′-CCTAYGGGRBGCASCAG-3′)/806R (5′-GGACTACNNGGGTATCTAAT-3′) (Berg et al., 2012) to determine the bacterial community structure and composition. Pyrosequencing was conducted on an Illumina (San Diego, CA, United States) HiSeq platform at Novogene (Beijing, China). Sequencing reads were assigned to samples based on their unique barcode. After cutting off the barcode and primer sequence, original sequences in each sample were assembled into raw tags using FLASH program (V1.2.7^4^) (Magoc and Salzberg, 2011). To obtain effective tags, raw tags were subjected to quality control (according to the QIIME V1.7.0) and the chimera sequences were removed referring to the previously researches (Edgar et al., 2011; Haas et al., 2011). Effective tag analysis was accomplished to calculate alpha and beta diversity by QIIME and Uparse software (Edgar, 2013). Sequences were assigned into operational taxonomic units (OTUs) at the threshold of 97% sequence similarity, as suggested previously (Stackebrandt and Goebel, 1994; Edgar, 2013), and the sequences were annotated using the GreenGene Database (Desantis et al., 2006). All of the sequences obtained in this study were deposited in the National Center for Biotechnology Information (NCBI) sequence read archive under the accession number SRP125984.

### Data Analysis

Correlation analysis was applied to determine the relationships of airborne bacterial characteristics with pollutant concentrations and meteorological parameters. Analysis of variance (ANOVA) and *t*-tests was performed to examine the significant differences among the variables. All these analyses were conducted using SPSS version 18.0 and *p*-values of <0.05 were defined as being statistically significantly different.

Principal component analysis (PCA) was applied to show the different microbial community structures among the samples. Redundant analysis (RDA) was applied to assess the relationships between biological features and environment parameters. Analysis of similarity (ANOSIM) was used to test the differences in microbial community structures among distinct groups. These analyses were conducted using the Vegan package (v.2.2-1) in the R computing environment. The linear discriminant analysis (LDA) effect size (LEfSe) method was applied to explore the bacterial markers distinguishing between the haze and no-haze samples. This analysis was conducted within the Galaxy web application and workflow framework^5^. The Kruskal–Wallis rank sum test was implemented to find biomarkers with significantly different (*p* < 0.05) relative abundance among the classes.

## Results

### Staged Characteristics of Haze Events at Environmental Factors

In our study, bioaerosols were sampled from nine independent haze events (Nos. 1–9) that occurred over different seasons (including the winter heating season) and that presented distinct temperatures and concentrations of SO_2_ (Supplementary Figure S3). Because of changes in the emissions scenario, the SO_2_ concentration during the winter heating season was higher than that in the non-heating season during the haze days, which was similar to previously reported data (Wang et al., 2015). Additionally, beginning in the middle of December, the average temperature remained below 0°C in Beijing. Thus, according to temperature and concentration of SO_2_, the nine haze events were classified into three stages: Stage I (Nos. 1–3 haze events from October 1 to November 6, 2015) corresponding to the non-heating season; Stage II (Nos. 4–6 from November 25 to December 15, 2015) belonging to the heating season with average temperature above 0°C; and Stage III (Nos. 7–9 from December 18, 2015, to January 5, 2016) belonging to the heating season, with an average temperature lower than 0°C.

### The Variation in Airborne Bacterial Concentration During the Haze Process

**FIGURE 1:**
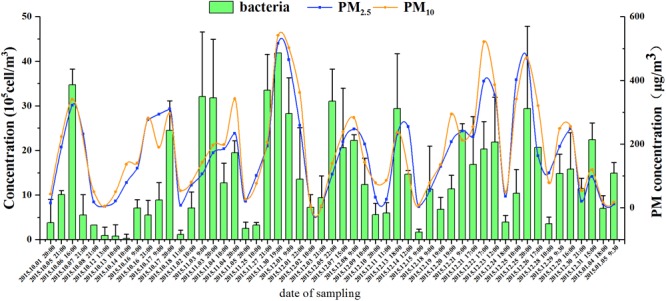
The concentration of airborne bacteria, PM_2.5_, and PM_10_ during nine haze events from October 1, 2015, to January 5, 2016. Error bars represent SD of samples from non-haze or haze days in each haze event, respectively.

Generally, *C*_ab_ increased for the haze days and showed cyclical changes consistent with the haze process (**Figure 1**). Taking the first (No. 1) haze process as an example (October 1–8, 2015), the concentration of PM_2.5_ was 15 μg/m^3^ at 20:00 on October 1 when the pollution level was clean; it increased to 191 and 322 μg/m^3^ at 21:00 on October 5 and 16:00 on October 6, respectively, with a concomitant deterioration in air quality; finally, it declined to 232 μg/m^3^ at 21:00 on October 7 as winter northerly winds increased and eventually decreased to a clean level of 18 μg/m^3^ at 21:00 on October 8. Corresponding to the haze cycle, the *C*_ab_ concentrations were 3.81 (±3.77) × 10^5^, 1.01 (±0.09) × 10^6^, 3.47 (±0.36) × 10^6^, 5.50 (±4.61) × 10^5^, and 3.27 (±0.00) × 10^5^ cells/m^3^, respectively, in this haze cycle. *C*_ab_ increased significantly by one order of magnitude for the haze days compared with the non-haze days (ANOVA, *p* < 0.05). In all nine haze events, the *C*_ab_ also showed the same dynamic variation and was positively correlated with the concentration of the main pollutants (PM_2.5_, PM_10_, SO_2_, NO_2_, and CO) and RH (Spearman’s correlation, *p* < 0.05, Supplementary Table S1). These data clearly demonstrated the positively correlated cycling features of the pollution level and the *C*_ab_.

**FIGURE 2:**
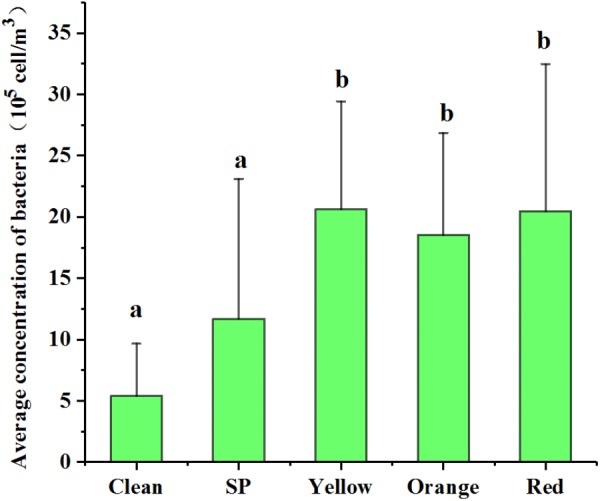
Variation of bacterial concentration during different haze pollution levels. Clean, SP (Slightly Polluted), yellow, orange, and red represent the haze pollution levels from non-haze to severe haze. Statistical significance is indicated by different letters (a, b) in columns as assessed by ANOVA (*p* < 0.05).

When the samples were grouped according to their pollution levels, the averages of *C*_ab_ at yellow, orange, and red levels (2.06 × 10^6^, 1.85 × 10^6^, and 2.05 × 10^6^ cells/m^3^, respectively) were significantly higher (ANOVA, *p* < 0.05) than those at the clean and SP levels (5.41 × 10^5^ and 1.17 × 10^6^ cells/m^3^, respectively) (**Figure 2**). However, the averages of *C*_ab_ among yellow, orange, and red levels were not significantly different. These results indicated that the airborne bacterial concentration increased remarkably from non-haze to haze pollution and plateaued at the “yellow haze alarm.”

**FIGURE 3:**
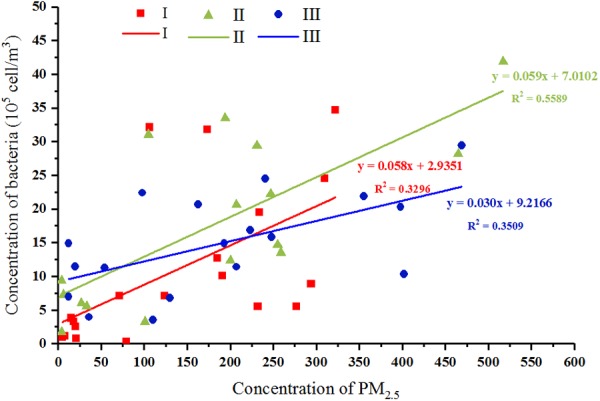
Correlations between bacterial concentration and PM_2.5_ mass concentration at three stages, respectively. Red, green, and blue represent samples collected from stages I, II, and III, respectively. The linear model between *C*_ab_ and PM_2.5_ index were shown.

At the three sampling stages, a minimum *C*_ab_ of 2.72 × 10^4^, 1.69 × 10^5^, and 3.54 × 10^5^ cells/m^3^ and a maximum *C*_ab_ of 3.47 × 10^6^, 4.19 × 10^6^, and 2.94 × 10^6^ cells/m^3^ were, respectively, observed. Haze and non-haze samples at Stage III showed the smallest *C*_ab_ variation but a similar PM_2.5_ amplitude to those at Stages I and II. Meanwhile, although *C*_ab_ was positively correlated with the PM_2.5_ index at all three stages (*p* < 0.05, Supplementary Table S2), their correlation coefficients were similar at Stage I and Stage II (r = 0.697^∗∗^, 0.687^∗∗^, Supplementary Table S2), which were both greater than that at Stage III (*r* = 0.495^∗^, Supplementary Table S2). The linear model between *C*_ab_ and PM_2.5_ index also showed a consistent result (**Figure 3**). These results might imply that the effects of haze pollution on airborne bacterial concentration at Stages I and II were significantly greater than that at Stage III.

### Key Environmental Factors Affecting the Airborne Bacterial Community Structure

For community structure analysis, a total of 46 samples, including haze and non-haze samples of the nine haze events, were sequenced on an Illumina HiSeq platform for the 16S rRNA gene. After quality control, a total of 1,861,965 clean reads were obtained and assigned to 1,294 OTUs at 97% similarity. The number of reads varied from 21,632 to 57,106 and the number of OTUs varied from 1,083 to 3,463 in each of the samples. The refraction curves (Supplementary Figure S4) demonstrated that most of the OTUs have been recovered.

**FIGURE 4:**
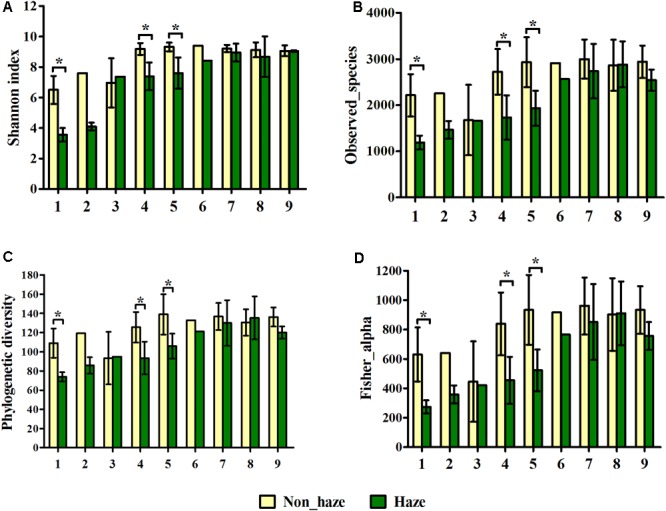
Shannon index **(A)**, observed species **(B)**, phylogenetic diversity **(C)** and fisher alpha **(D)** of bacterial community on haze and non-haze days in nine haze events during October 1, 2015, to January 5, 2016. Nos. 1–9 represent nine haze events. Yellow and green columns represent samples from non-haze and haze days, respectively. Error bars represent SD of samples from non-haze or haze days in each haze event, respectively. Non-haze samples in No. 2 and No. 6, and haze samples in No. 3 and No. 6, were not repeated because haze duration was too short in these haze events. *T*-test, ^∗^*p* < 0.05.

All the alpha diversity indices, including Shannon index, observed species, phylogenetic diversity (PD) index, and the fisher index were lower in the haze day samples compared with those in the non-haze days, although significant differences were only observed in the haze samples for No. 1, No. 4, and No. 5 haze events (*t*-test, *p* < 0.05) (**Figure 4**). Pearson correlation coefficients between the alpha diversity indices and environmental factors also showed the same trend (Supplementary Table S3). Observed species, PD index, and fisher index had a significant negative relationship with the concentration of PM_2.5_, PM_10_, and RH (Supplementary Table S3). Notably, alpha diversity of haze samples at Stage III showed no an obvious decrease compared with that of the non-haze samples (**Figure 4**), which was different from the change of alpha diversity at Stages I and II. This observation was consistent with the airborne bacterial concentration changes mentioned previously. Both of these findings might imply that the haze events at Stage III had only slight effects on airborne bacteria. In addition, significant correlations of alpha diversity with AP and between alpha diversity and T were detected (Supplementary Table S3), which implied that meteorological factors had important effects on airborne microbial community structure. These results suggested that haze pollution could decrease the alpha diversity in the bioaerosols, and this effect was weaker after midwinter.

**FIGURE 5:**
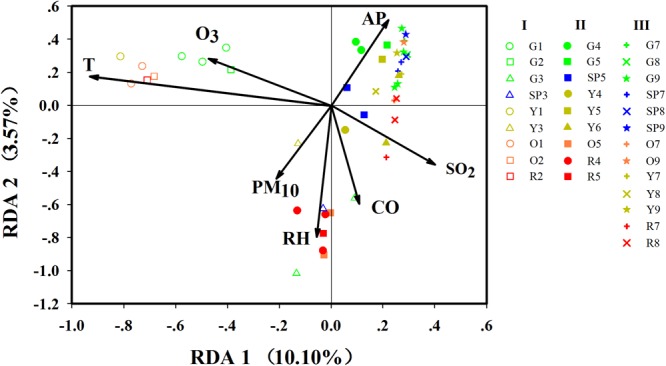
Redundancy analysis (RDA) of biological with environmental parameters. G, SP, Y, O, and R, respectively, represent haze pollution levels green, slightly polluted, yellow, orange and red. Nos. 1–9 represent the nine haze events during October 1, 2015, to January 5, 2016. Only significant environmental variables are shown in this figure, while detailed information on environmental variables are shown in Supplementary Table S5.

Airborne bacterial community structure had obvious differences among the three stages (ANOSIM, *p* < 0.01, Supplementary Table S4) and was significantly affected by temperature (*r*^2^ = 0.8043, *p* = 0.001) and SO_2_ concentration (*r*^2^ = 0.2245, *p* = 0.002) (Supplementary Table S5). Redundancy analysis of genus-level taxonomic composition with environmental factors (**Figure 5**) suggested that bacterial composition in Stage I samples was positively correlated with temperature but negatively correlated with the concentration of SO_2_, whereas Stages II and III samples showed opposite features. This was consistent with the environmental conditions of each stage. At Stage I, the temperature was higher and the concentration of SO_2_ was lower than those at Stages II and III (Supplementary Figure S3). In addition, both the PCA plot and ANOSIM tests demonstrated that variations in the airborne bacterial community structure between stages were greater than variations between haze and non-haze samples (Supplementary Figure S5 and Supplementary Table S4). Thus, it made more sense to analyze airborne bacterial changes on haze and non-haze days based on various stages.

At Stage I, the samples on haze days were remarkably separated from the samples on non-haze haze days (ANOSIM, *r* = 0.2791, *p* < 0.05). At Stage II, samples showed similar profiles (ANOSIM, *r* = 0.3580, *p* < 0.05). Unexpectedly, at Stage III, the bacterial community structure varied slightly between haze and non-haze samples (ANOSIM, *r* = 0.1111, *p* < 0.05), which also was consistent with the results mentioned earlier that bacterial concentration and alpha diversity remained stable during the haze events at Stage III. RDA further indicated that RH (*r*^2^ = 0.3775, *p* = 0.001) and PM_10_ (*r*^2^ = 0.1516, *p* = 0.022) showed a positive correlation with the haze samples (**Figure 5**). Meanwhile, the haze samples at Stage II also were positively correlated with CO concentration (*r*^2^ = 0.2232, *p* = 0.005). Three independent RDAs analyses based on data from the three stages showed consistent results that RH, PM_10_, and CO were major environmental factors shaping the airborne bacterial community structure during haze events (Supplementary Figure S6).

### The Effects of Haze Events on Airborne Bacterial Composition

**FIGURE 6:**
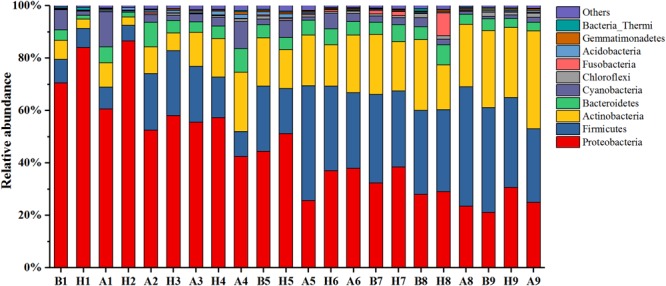
Bacterial community compositions at the phyla level during Nos. 1–9 haze events. Only the top 10 most abundant phyla are presented. The category “others” contains all the remaining bacterial phyla. “H” represents haze pollution samples, while “B” and “A” represent the non-haze samples collected “before” or “after” occurrence of haze.

In this study, 43 phyla covering 120 classes, 220 orders, 324 families, and 671 genera were detected across all samples. The relative abundance of the top 10 phyla is shown in **Figure 6**, and the sum of their relative abundance exceeded 99%. Proteobacteria, Firmicutes, and Actinobacteria were the predominant phyla in both the haze and non-haze samples. In most cases (except for the No. 6 haze samples), the relative abundance of Proteobacteria increased significantly (from 41.85 to 54.37% on average), whereas Actinobacteria decreased (from 19.88 to 13.48% on average) on the haze days, and no regular trend was observed for Firmicutes. Further analyses demonstrated that the variations in relative abundance of Proteobacteria and Actinobacteria were mainly caused by γ-proteobacteria and Actinobacteria, respectively. Pearson correlation analysis demonstrated that γ-proteobacteria were positively correlated with RH and CO (0.551^∗∗^, 0.321^∗^) and Actinobacteria were negatively correlated with temperature, PM_2.5_, PM_10_, and RH (-0.633^∗∗^, -0.318^∗^, -0.336^∗^, -0.372^∗^). These results demonstrated that PM_2.5_, PM_10_, CO index, and RH had obvious influences on airborne bacterial composition during the haze events, which also indicated that meteorological factors had significant effects on the community composition of airborne bacteria.

**FIGURE 7:**
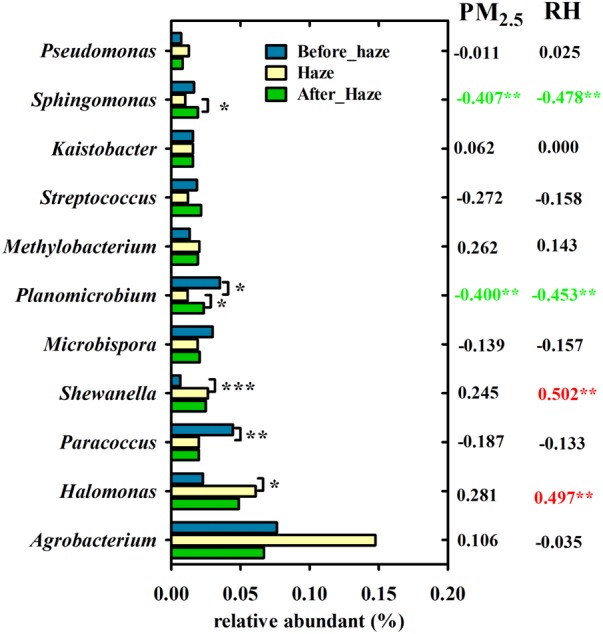
Variation in bacterial genera with ≥1% relative abundance among haze days, before and after haze pollution. Pearson’s correlation coefficients between these genera and PM_2.5_ index, RH is shown in the right. ^∗∗∗^*P* < 0.001, ^∗∗^*P* < 0.01, ^∗^*P* < 0.05.

At the genus level, *Agrobacterium, Halomonas, Paracoccus, Shewanella, Microbispora, Planomicrobium, Methylobacterium, Streptococcus, Kaistobacter, Sphingomonas*, and *Pseudomonas* were predominant with average relative abundances exceeding 1%. As shown in **Figure 7**, the relative abundance of *Halomonas* and *Shewanella* in the haze samples (6.10 and 2.64%, respectively) was significantly greater than that in the samples collected before haze developed (2.29 and 0.66%, respectively, *t*-test). Meanwhile, they were positively correlated with RH (0.497^∗∗^ and 0.502^∗∗^, respectively). The relative abundance of *Paracoccus, Planomicrobium*, and *Sphingomonas* was significantly decreased on the haze days (*t*-test) and, except for *Paracoccus*, were significantly negatively correlated with PM_2.5_ and RH. These results not only indicated that *Halomonas* and *Shewanella* (γ-proteobacteria) were enriched on the haze days but also that PM and RH were the main factors affecting airborne bacterial composition.

### The Variations in Airborne Bacterial Composition During Haze Events at Distinct Stages

As shown in **Figure 6**, although Proteobacteria, Firmicutes, and Actinobacteria were predominant phyla in all samples, their relative abundances showed obvious seasonal changes. As the temperature decreased over time, from October 1, 2015, to January 5, 2016, the relative abundance of Proteobacteria gradually decreased, too. Firmicutes and Actinobacteria, however, gradually increased. Pearson correlation analysis displayed the same results, where temperature was positively correlated with Proteobacteria (0.738^∗∗^) but negatively correlated with Firmicutes and Actinobacteria (-0.544^∗∗^, -0.650^∗∗^). Because of the seasonal distinction of predominant bacteria, the characteristics of bacterial changes at distinct stages should be examined in other haze studies.

**FIGURE 8:**
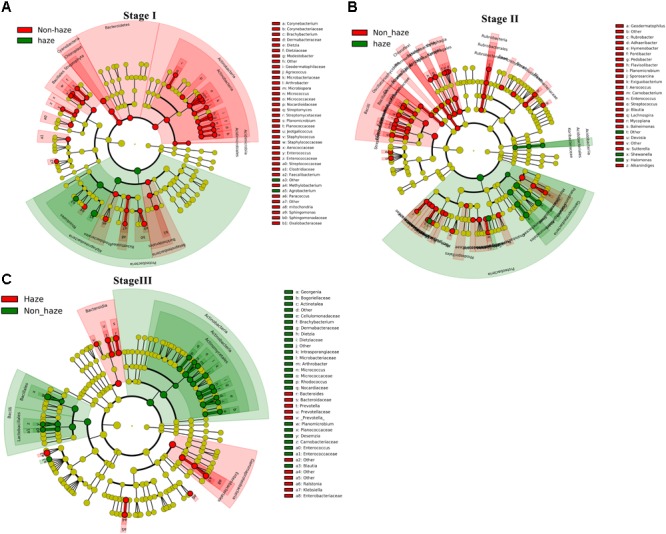
Discriminant genera between samples collected from the haze and non-haze days at Stage I **(A)**, Stage II **(B)**, and Stage III **(C)** estimated by LEfSe analysis based on the top 0.1% relative abundance in all samples. Red and green colors represent taxa with significant changes on the haze and non-haze days, respectively (yellow, not significant). The size of each circle is proportionate to the taxon’s relative abundance. Only taxa with an LDA score >2.0 are shown.

To explore the bacterial changes at each stage, bacterial genera with relative abundance exceeding 0.1% were analyzed using LEfSe analysis. As shown in **Figure 8**, the relative abundance of *Agrobacterium* increased remarkably during the haze events at Stage I. The relative abundance of *Halomonas* and *Shewanella*, which were positively correlated with RH, showed a significant increase on the haze days at Stage II. Although the bacterial concentration, alpha diversity, and community structure were only slightly affected by the haze events at Stage III, the relative abundances of *Klebsiella, Ralstonia, Prevotella*, and *Bacteroides* increased significantly at this stage.

## Discussion

The sharp rise in the concentration of PM is an obvious characteristic of haze events (Huang et al., 2014; Wang et al., 2016). To date, chemical composition, source, and statistical correlation with human health of non-biological particles have been widely studied during haze events (Huang et al., 2014, 2016; Wang et al., 2015, 2016; Guan et al., 2016; Zheng et al., 2016). The potential harm of biological particles and their relationships to climatic changes during these haze events, however, remain unclear. In the present study, we investigated variations in the abundance and the community composition of airborne bacteria during nine haze events to provide fundamental information about the relationship between bioaerosols and air pollution. Our results indicated that these haze events had significant effects on airborne bacterial concentration, community structure, and composition. In addition, we initially discovered an obvious staged distinction of these effects by analyzing samples from nine haze events occurring between October 1, 2015, and January 5, 2016.

In Beijing, haze pollution occurs frequently between October and March, corresponding to the late fall, winter, and early spring seasons. Additionally, this period also covers a special period (i.e., the winter heating season) in northern China, in which the chemical composition of PM is different from that in the non-heating season (Zhang et al., 2016, 2017; Liao et al., 2017). On the basis of differences in temperature and chemical composition of PM during haze events, in this study, the nine haze events were classified into three stages for comparative analysis. As expected, the airborne bacterial community structure showed significant differences among the three stages, which was consistent with previous reports that airborne bacterial concentration and community composition were shaped by meteorological factors (Franzetti et al., 2011; Yamamoto et al., 2012; Bowers et al., 2013; Qi et al., 2014; Gao et al., 2016; Du et al., 2017). Most notably, our study also revealed that the effects of haze events on airborne bacteria were slighter than the effects of temperature (**Figure 5**) and exhibited differences between fall and winter (**Figures 3, 5**).

Most of the previous studies focused on haze events that occur in January (the month in which these haze events occur most frequently and are the most severe) and demonstrated that the predominant bacterial community structure remained stable on both the haze and non-haze days (Cao et al., 2014; Wei et al., 2016; Du et al., 2017). These previous results were further confirmed by our findings in airborne bacterial concentration, alpha diversity, and community structure during the same time period (Stage III: December 18, 2015, to January 5, 2016). The data from the other two stages in our study, however, showed different results. Increased airborne bacterial concentration, decreased alpha diversity, and significantly changed community structure were observed on the haze days compared with the non-haze days at Stage I (October 1 to November 6, 2015) and Stage II (November 25 to December 15, 2015). Formation of haze relies on stagnant atmospheric conditions (Cai et al., 2017), which are not conducive to PM dilution (Ma et al., 2016). Meanwhile, one of the states of bacteria in the atmosphere is to adhere to PM (Smets et al., 2016; Zhai et al., 2018). Therefore, haze events also are not conducive to airborne bacteria dilution. Because more bacteria might be suspended in the atmosphere, airborne bacterial concentration increased and community structure changed during the haze events. These variations were obvious at Stages I and II (**Figures 3–5**). At Stage III, however, the average temperature was below zero (Supplementary Figure S3), which resulted in ground frost. This condition may not be conducive to the suspension of terrestrial bacteria in the atmosphere. Cao et al. (2014) reported that terrestrial bacteria were predominant airborne bacteria in the atmosphere during haze events. Haze events under this condition did not cause more airborne bacteria suspended in the atmosphere to change airborne bacterial concentration and community structure. Therefore, during haze events at Stage III, airborne bacterial concentration and community structure remained stable, which was consistent with previous studies (Cao et al., 2014; Wei et al., 2016; Du et al., 2017). Our study, for the first time, clarified that the changes in bioaerosols during the haze events were a season-dependent feature.

It is critical to identify the key environmental factors that affect airborne bacterial characteristics in haze events. In the present study, PM_10_ and RH were the most significant factors associated with airborne bacterial concentration and community structure on the haze and non-haze days. Similar to previously reported data (Alghamdi et al., 2014; Dong et al., 2016), airborne bacterial concentration was significantly and positively correlated with the concentration of PM_10_ (Supplementary Table S1). Moreover, our study also indicated that PM_10_ significantly affected the community structure of airborne bacteria (**Figure 5** and Supplementary Table S3). As described in the previous paragraph, PM can serve as a carrier for airborne bacteria (Smets et al., 2016; Zhai et al., 2018). Meanwhile, the concentration PM was enhanced during haze events (Ma et al., 2016). Thus, during haze events, PM_10_ was a dominant factor that significantly affected airborne bacteria. RH was another contributing factor, which had a significant and positive effect on airborne bacterial concentration and community structure. The RH of the atmosphere was nearly equilibrated with the water activity in PM (Dannemiller et al., 2017), which is vital for airborne microbial survival and can shape community structure (Zhen et al., 2017). Dannemiller et al. (2017) reported that an elevated RH could provide comfortable conditions for airborne microbial survival, especially when the RH was higher than 80%. Elevated RH is another feature of haze events (Sun et al., 2013; Quan et al., 2014; Wang et al., 2016). In the present study, at orange and red pollution levels, the average RH reached 81 and 95%, respectively (Supplementary Figures S1, S2), which were beneficial to the survival of airborne bacteria.

Some studies have evidenced that *C*_ab_ is positively correlated with the PM index during the haze events (Dong et al., 2016; Wei et al., 2016). In the present study, comparable results were observed in the nine haze events over a long-term period. Stability in *C*_ab_ also was observed among the samples, however, at yellow, orange, and red levels, which might be explained by the higher concentrations of SO_2_ and NO_2_ at severe haze pollution levels. Xu et al. (2017) reported that airborne bacterial concentration decreased and was negatively correlated with the concentrations of SO_2_ and NO_2_. In their study, PM concentrations were similar to those found in our study, but the maximum concentration of SO_2_ (more than 300 ppb) was far higher than that in the studies of Dong et al. (2016) and Wei et al. (2016) as well as those found in our study (65.9 μg/m^3^, 80 ppb, and 68 μg/m^3^, respectively). This result suggested that *C*_ab_ was mainly affected by the PM but also was regulated by other pollutants, such as SO_2_.

The knowledge accumulated on the effect of haze on airborne bacterial community composition is limited. By comparing samples from haze and non-haze days in nine haze cycles, it can be suggested that Proteobacteria, especially γ-proteobacteria, was enriched on the haze days. Moreover, the γ-proteobacteria genera *Halomonas* and *Shewanella* were enriched in seven of the nine haze events, and their average relative abundances were significantly and positively correlated with RH. *Halomonas* mainly are isolated from highly saline environments, such as oceans and salt lakes (Aguirre-Garrido et al., 2016; Li Z. et al., 2016), alkaline-saline soils (Dou et al., 2015), and dumps polluted by solid wastes (Mwaikono et al., 2016). These organisms have great tolerance for high concentrations of nitrate and salinity (Liao et al., 2015). *Shewanella* have been reported as marine bacteria but also can be isolated from soils (Ye et al., 2016). Both *Halomonas* and *Shewanella* are highly tolerant to heavy metals (Shen et al., 2016). Moreover, they can detoxify arsenic (As), cadmium (Cd), and chromium (Cr) in the contaminated environments (Watts et al., 2015; Jain et al., 2016; Li C. et al., 2016). These two bacteria in the haze-polluted air of Beijing might originate from the alkaline-saline soils around Beijing, and their relative proportional increases in hazes might be related to their resistance in conditions of high pollutant concentrations and RH. This was the first report to show that *Halomonas* and *Shewanella* were dominant and significantly enriched bacteria during the haze events. In addition, *Klebsiella* was enriched on haze days at Stage III, which was detected previously as a bacterial pathogen in PM (Liu et al., 2017). The enrichment of pathogenic bacteria related to human diseases on the haze days deserves public health attention and further study.

Conclusively, in the present study, the effects of haze on airborne bacterial abundance and community composition were obviously distinct during different stages of haze events in Beijing. The correlations between haze pollution and airborne bacterial characteristics in midwinter (December 18, 2015 to January 5, 2016) were weaker than those in the fall and early winter (October 1 to December 15, 2015). In addition, *C*_ab_ showed significant cyclical positive correlations with the development of haze. Nonetheless, when haze became severe (pollution levels at yellow, orange, and red), *C*_ab_ did not concomitantly increase with pollutant concentration increases. Moreover, PM_10_ and RH were the key factors that significantly affected the airborne bacterial concentration and community structure. Finally, *Halomonas* and *Shewanella* were enriched on haze days, and they were positively correlated with RH. These results systematically elucidated airborne bacterial variations and revealed key environmental factors affecting airborne bacteria during haze events.

## Author Contributions

WL and HY conceived and designed the experiments. WL performed all the experiments and analysis. JY and EW discussed the results on microbiology ecology. DZ discussed the results on atmospheric science. BL participated in the air sample collection. WL wrote the paper. HY supervised the overall work, discussed the results, and revised the manuscript. All authors read and approved the final version of the manuscript.

## Conflict of Interest Statement

The authors declare that the research was conducted in the absence of any commercial or financial relationships that could be construed as a potential conflict of interest.

## Footnotes

1 ^1^http://www.bjmemc.com.cn/

2 ^2^http://www.weather.com.cn/

3 ^3^http://www.nmc.cn/

4 ^4^http://ccb.jhu.edu/software/FLASH/

5 ^5^http://huttenhower.sph.harvard.edu/galaxy/

## Abbreviations

*C*_ab_: the concentration of airborne bacteria
PM: particulate matter
