# Supplementary material for: Concentration and Community of Airborne Bacteria in Response to Cyclical Haze Events During the Fall and Midwinter in Beijing, China
Source: Front Microbiol. 2018 Jul 31;9:1741. doi: 10.3389/fmicb.2018.01741 (PMC6079307; doi:10.3389/fmicb.2018.01741)
Supplement: Supplementary file 1 [file Presentation_1.pdf]

Supplementary Material

Concentration and community composition of airborne bacteria staged responding to the haze events in Beijing, China

Weilin Li[1], Jinshui Yang[1], Daizhou Zhang[2], Baozhen Li[1], Entao Wang[3], Hongli Yuan1,*

*** Correspondence:**

Corresponding Author: Hongli Yuan;

E-mail address: hlyuan@cau.edu.cn

Supplementary Figures and Tables

1.1 Supplementary Figures

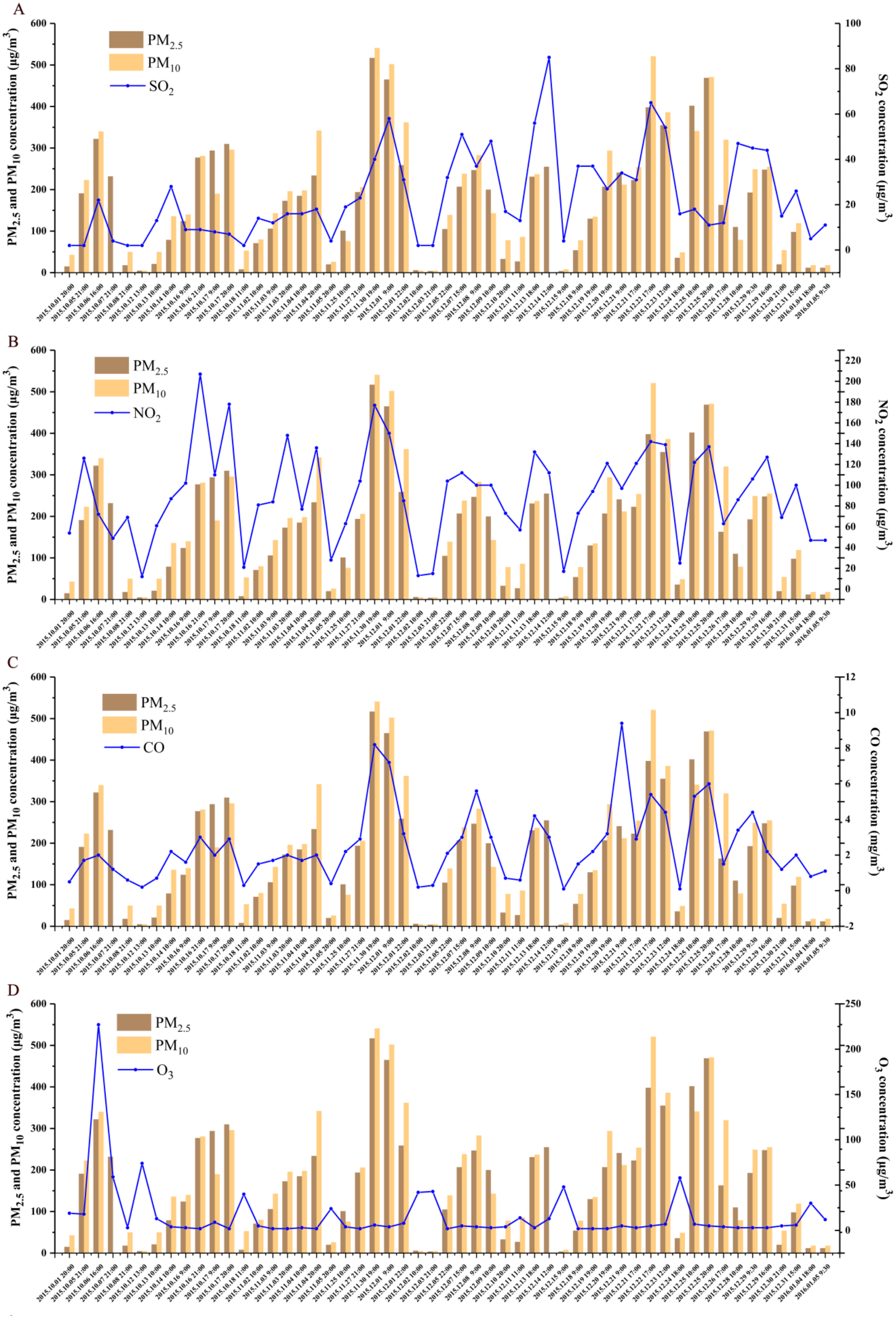

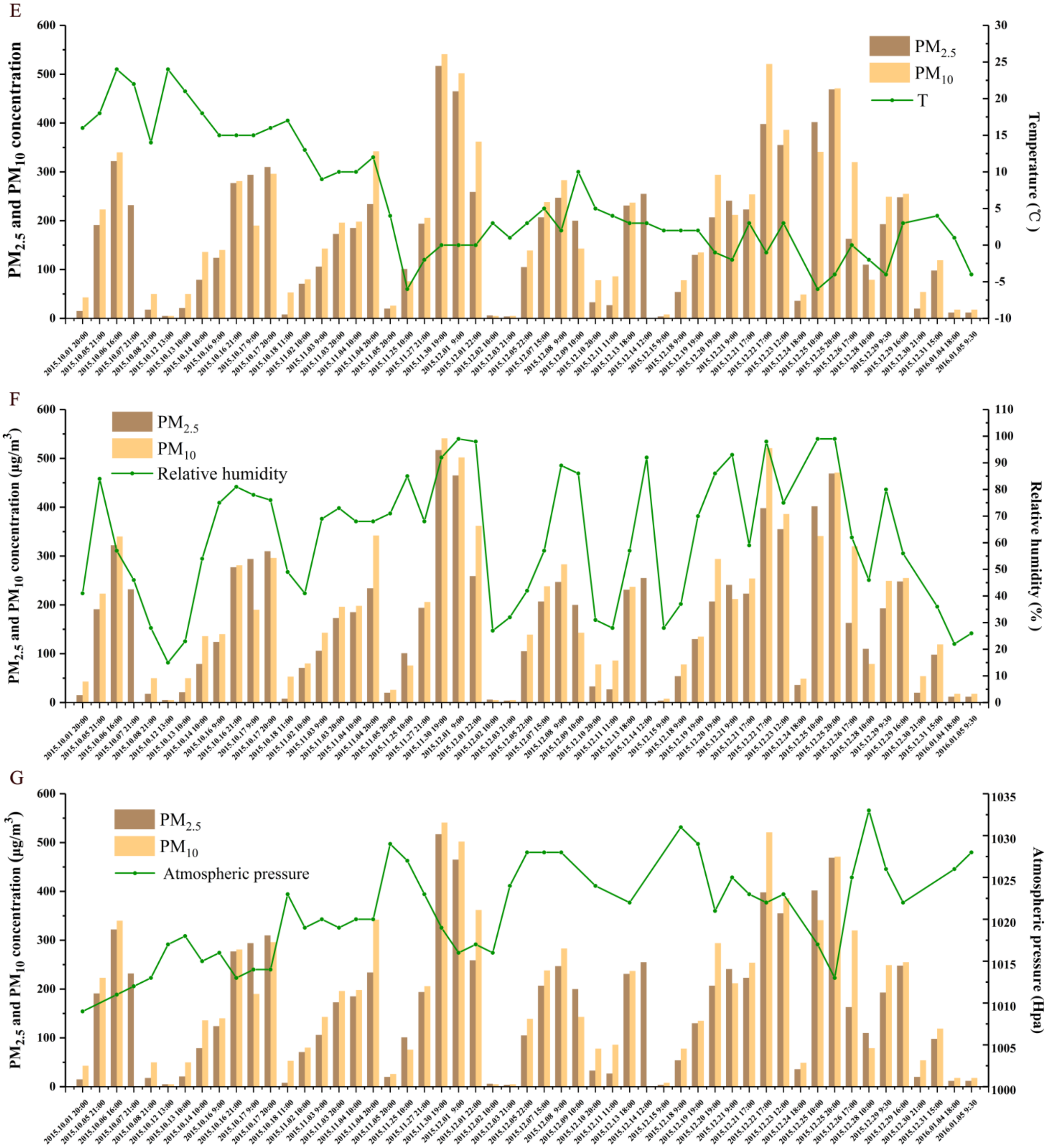

Supplementary Figure 1. Pollutant concentrations and meteorological data when samples were collected to enumerate airborne bacterial concentration. (A) SO_2 concentration, (B) NO_2 concentration, (C) CO concentration, (D) O_3 concentration, (E) Temperature, (F) Relative humidity, and (G) atmospheric pressure.

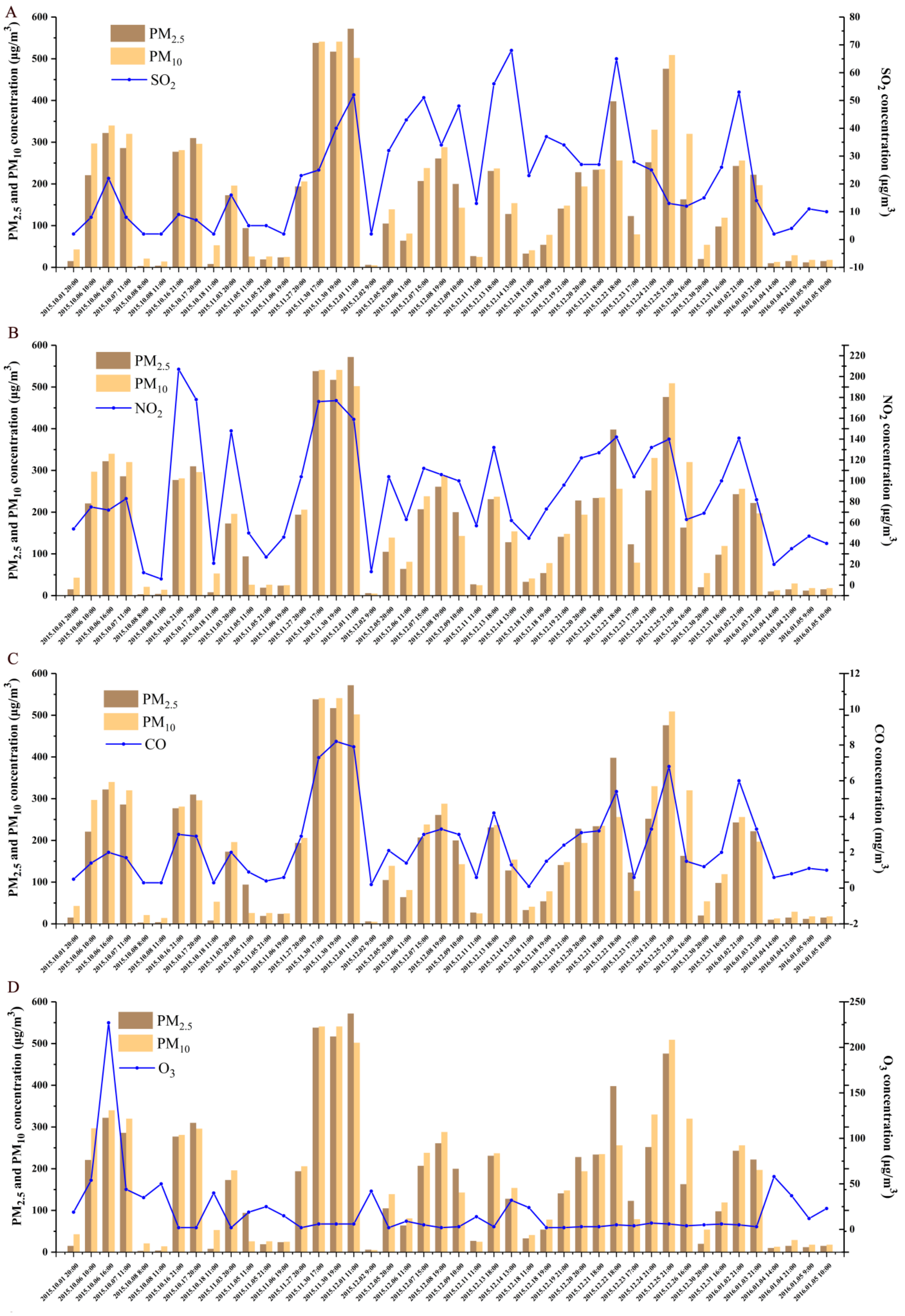

A
PM2.5
PM10
SO2
PM2.5 and PM10 concentration (μg/m3)
SO2 concentration (μg/m3)
B
PM2.5
PM10
NO2
PM2.5 and PM10 concentration (μg/m3)
NO2 concentration (μg/m3)
C
PM2.5
PM10
CO
PM2.5 and PM10 concentration (μg/m3)
CO concentration (mg/m3)
D
PM2.5
PM10
O3
PM2.5 and PM10 concentration (μg/m3)
O3 concentration (μg/m3)

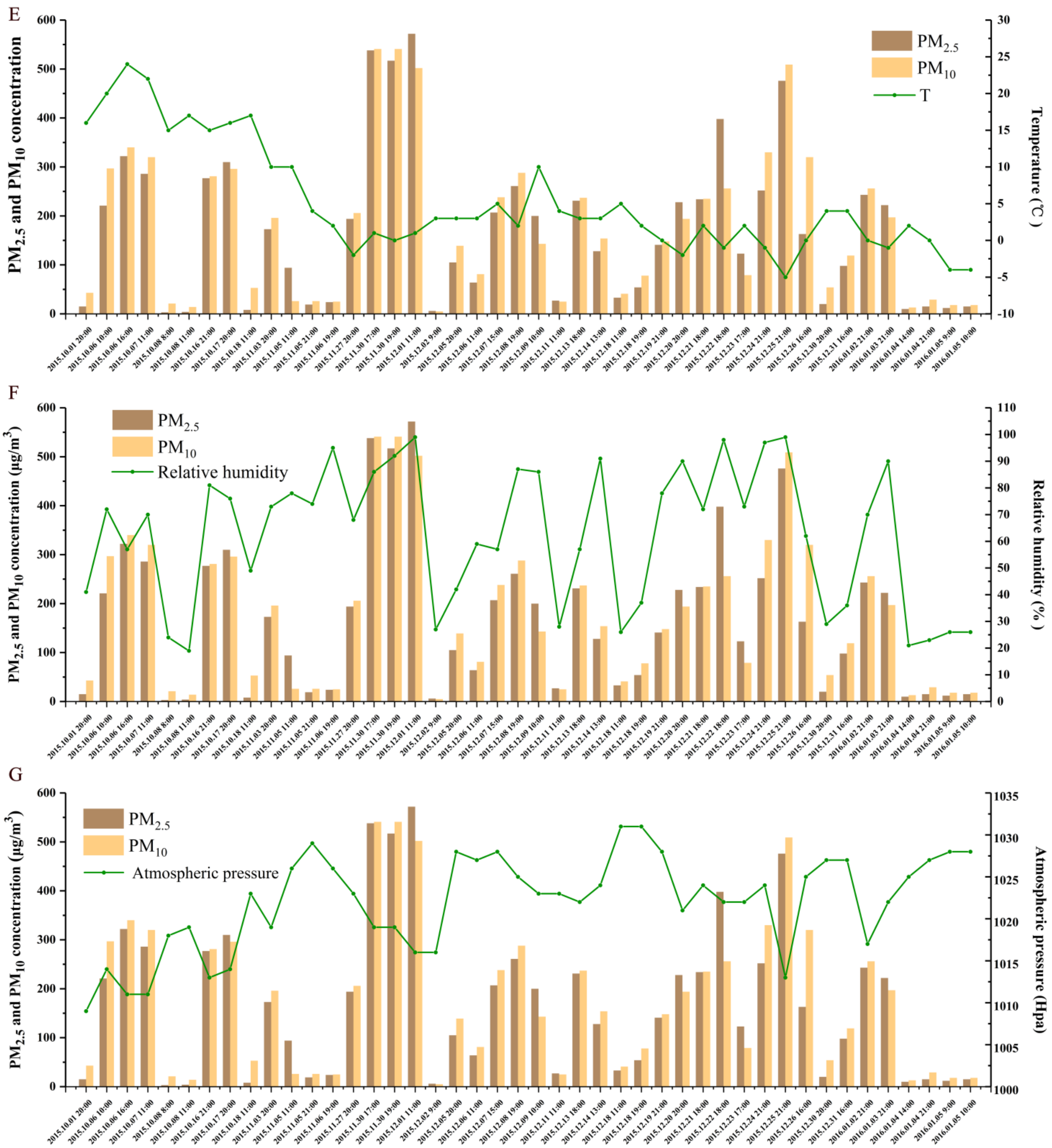

Supplementary Figure 2. Pollutant concentrations and meteorological data when samples were collected to analyze airborne bacterial community structure. (A) SO_2 concentration, (B) NO_2 concentration, (C) CO concentration, (D) O_3 concentration, (E) Temperature, (F) Relative humidity, and (G) atmospheric pressure.

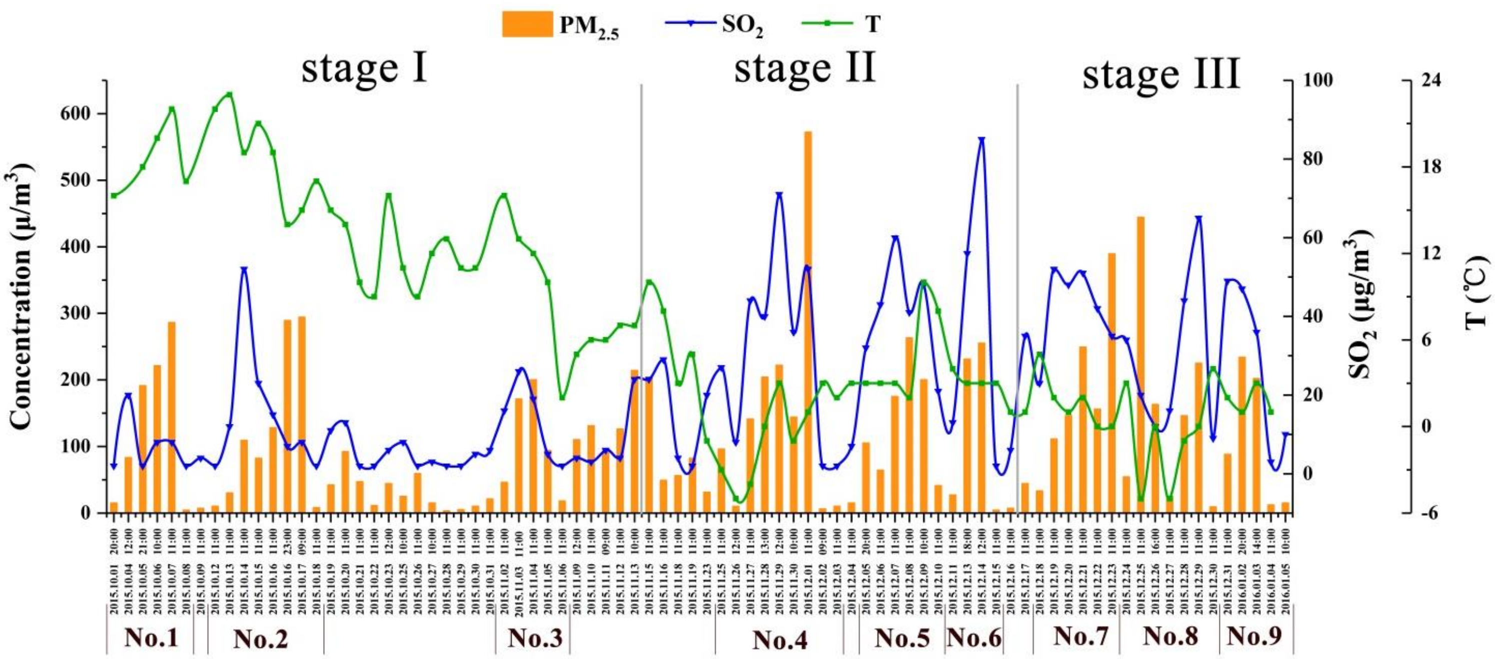

Supplementary Figure 3. Daily temperature and concentration of PM2.5 and SO2 during October 1st 2015 to January 5th 2016. During this period, nine independent haze events (No. 1-No. 9) occurred and were divided into three stages for comparison.

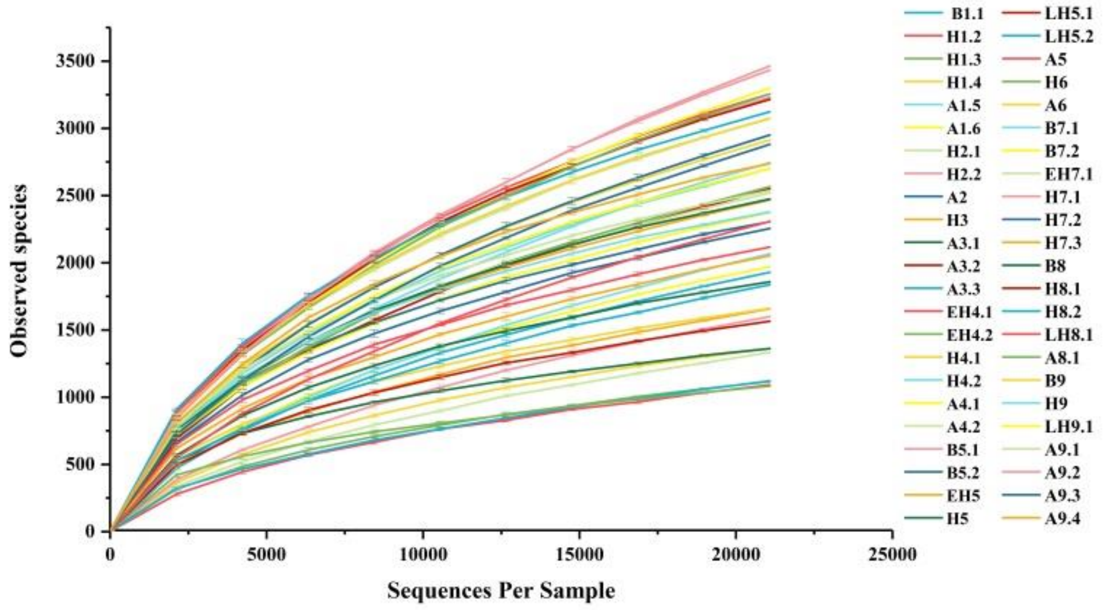

Supplementary Figure 4. Rarefaction curves of all the samples

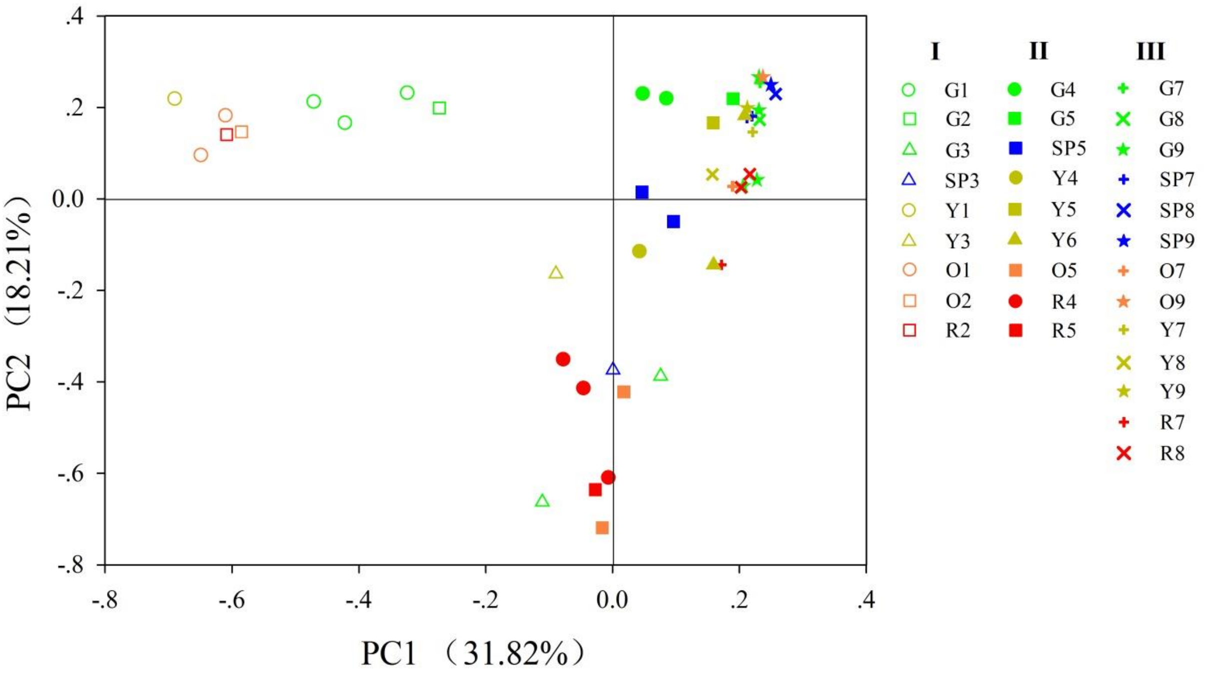

Supplementary Figure 5. Principal component analysis of the bacterial community. G, SP, Y, O, and R respectively represent haze pollution levels "Green", "Slightly Polluted", "Yellow", "Orange" and "Red". No.1-No.9 represent the nine haze events during Oct. 1st 2015 to Jan. 5th 2016.

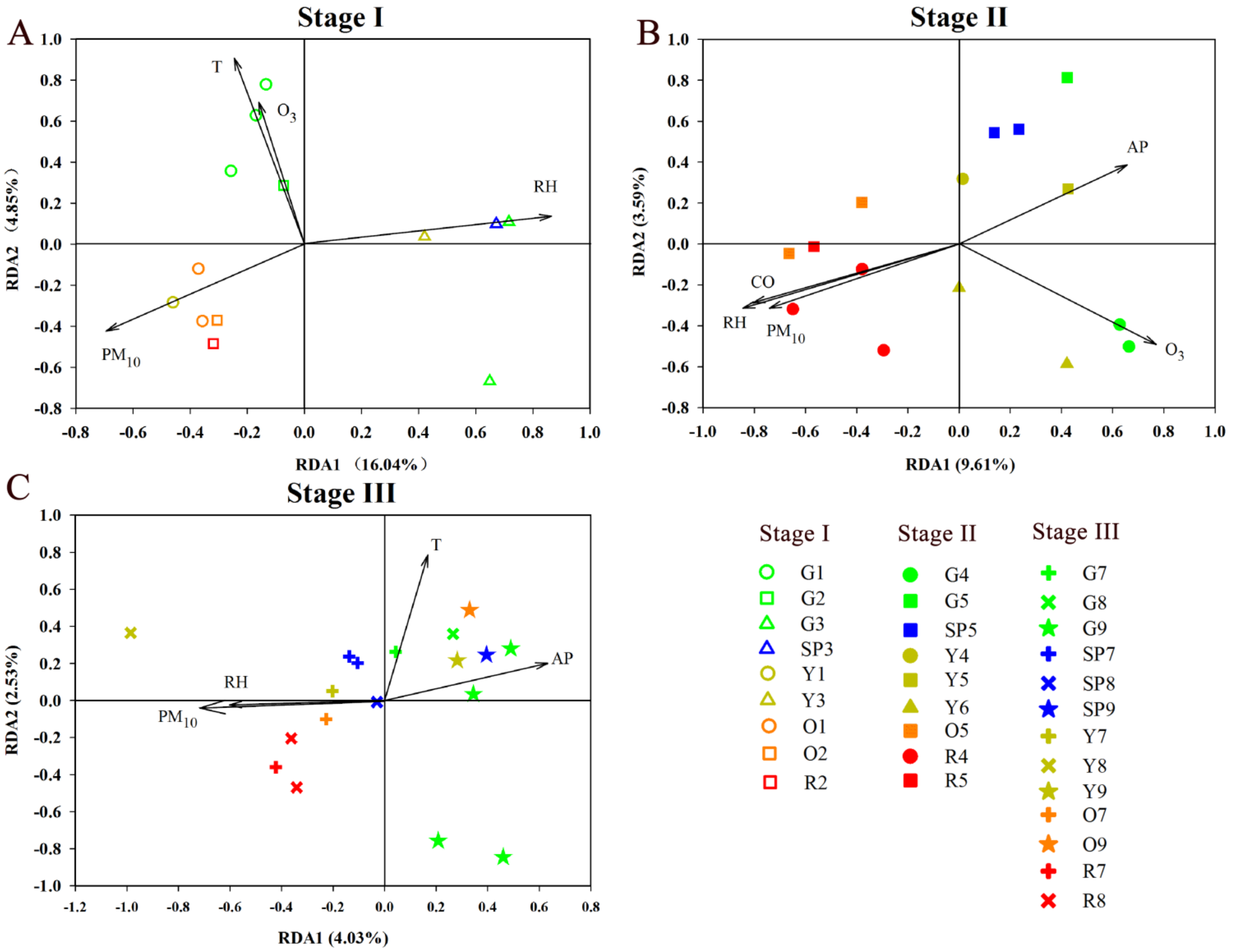

Supplementary Figure 6. Redundancy analysis (RDA) of biological with environmental parameters at Stage I (A), Stage II (B) and Stage III (C) independently. G, SP, Y, O, and R respectively represent haze pollution levels "Green", "Slightly Polluted", "Yellow", "Orange" and "Red". No. 1-No. 9 represent the nine haze events during Oct. 1st 2015 to Jan. 5th 2016. Only significant environmental variables are shown in this figure.

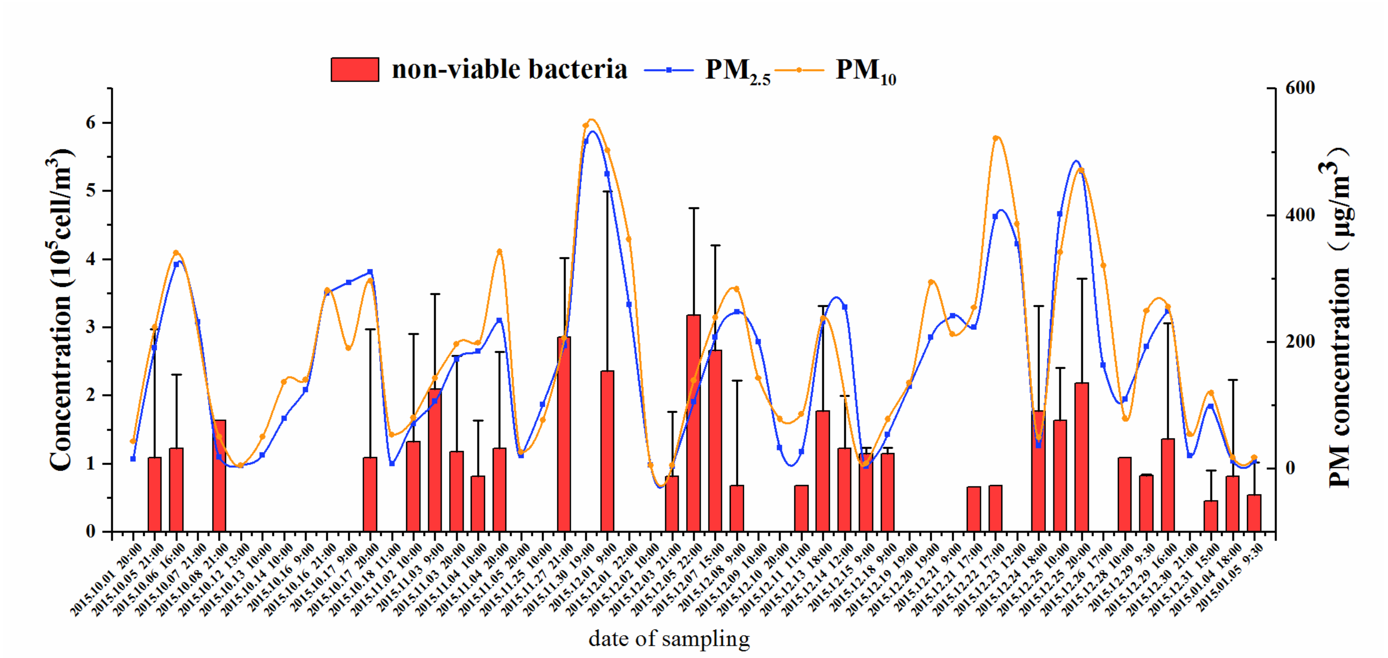

Supplementary Figure 7. The concentration of non-viable airborne bacteria, $PM_{2.5}$, and PM_{10} during nine haze events from October 1st 2015 to January 5th 2016. Error bars represent SD of samples from non-haze or haze days in each haze event, respectively.

1.2 Supplementary Tables

Supplementary Table 1. Spearman's correlation coefficients between airborne bacterial concentration and pollutants, meteorological parameters.

	$PM_{2.5}$	PM_{10}	SO_2	NO_2	O_3	CO	AP	T	RH	WS
C_{ab} [a]	0.624**	0.655**	0.447**	0.561**	-0.268	0.543**	-0.015	-0.297	0.421**	-0.340*

** P <0.01 (2-tailed), * P < 0.05 (2-tailed)

[a] C_{ab}: concentration of airborne bacteria.

Supplementary Table 2. Spearman's correlation coefficients between airborne bacterial concentration and pollutants, meteorological parameters at each stage, respectively.

	Concentration of airborne bacteria		
	Stage I	Stage II	Stage III
$PM_{2.5}$	0.697**	0.687**	0.495*

Supplementary Table 3. Pearson's correlation coefficients between pollutants, meteorological parameters and alpha diversity indexes.

	$PM_{2.5}$	PM_{10}	SO_2	NO_2	O_3	CO	AP	T	RH	WS
Shannon index	-0.281	-0.299*	0.244	-0.175	-0.390**	-0.021	0.620**	-0.763**	-0.241	0.223
observed_species	-0.376*	-0.345*	0.058	-0.252	-0.256	-0.185	0.468**	-0.565**	-0.397**	0.200
PD_whole_tree	-0.368*	-0.338*	0.071	-0.222	-0.283	-0.176	0.491**	-0.561**	-0.363*	0.148
fisher_alpha	-0.368*	-0.334*	0.040	-0.244	-0.237	-0.185	0.460**	-0.554**	-0.393**	0.202

** $p < 0.01$ (2-tailed), * $P < 0.05$ (2-tailed)

Supplementary Table 4. ANOSIM tests on the bacteria community between each stage and between haze & non-haze samples.

		R value	*P* value
stages	Stage I vs Stage II	0.4584	0.001
	Stage I vs Stage III	0.7677	0.001
	Stage II vs Stage III	0.3503	0.001
Haze vs Non-haze	Stage I	0.2791	0.030
	Stage II	0.3580	0.011
	Stage III	0.1111	0.039

Supplementary Table 5. Redundancy analysis (RDA) of bacterial community and environmental factors

	**RDA1**	**RDA2**	**r^2**	**Pr(>r)**
**T**	-0.98792	0.15494	0.8043	0.001***
**RH**	-0.0596	-0.99822	0.3775	0.001***
**O_3**	-0.92025	0.39133	0.2568	0.001***
**SO_2**	0.85398	-0.52031	0.2245	0.002**
**CO**	0.29844	-0.95443	0.2232	0.005**
**AP**	0.52018	0.85406	0.1975	0.010**
**PM_{10}**	-0.56326	-0.82628	0.1516	0.022*
**NO_2**	-0.16413	-0.98644	0.0984	0.112
**WS**	0.69831	0.7158	0.0709	0.204

*** $P < 0.001$, ** $P < 0.01$, * $P < 0.05$
